# Identification of a transient state during the acquisition of temozolomide resistance in glioblastoma

**DOI:** 10.1038/s41419-019-2200-2

**Published:** 2020-01-06

**Authors:** Marion Rabé, Solenne Dumont, Arturo Álvarez-Arenas, Hicham Janati, Juan Belmonte-Beitia, Gabriel F. Calvo, Christelle Thibault-Carpentier, Quentin Séry, Cynthia Chauvin, Noémie Joalland, Floriane Briand, Stéphanie Blandin, Emmanuel Scotet, Claire Pecqueur, Jean Clairambault, Lisa Oliver, Victor Perez-Garcia, Arulraj Nadaradjane, Pierre-François Cartron, Catherine Gratas, François M. Vallette

**Affiliations:** 1 CRCINA, INSERM, Université d’Angers, Université de Nantes, Nantes, France; 2 GenoBiRD, SFR François Bonamy, Université de Nantes, Nantes, France; 3 Department of Mathematics and MôLAB-Mathematical Oncology Laboratory, University of Castilla-la Mancha, Ciudad Real, Spain; 4 Laboratoire Jacques-Louis Lions, Inria, Mamba team and Sorbonne Université, Paris 6, UPMC, Paris, France; 5 IGBMC - CNRS UMR 7104-Inserm U 1258, Université de Strasbourg, 67404 Illkirch, France; 6 Laboratoire de Biologie des Cancers et Théranostic, Institut de Cancérologie de l’Ouest-St Herblain, 44805 Saint-Herblain, France; 7 Plate-Forme MicroPICell, SFR François Bonamy, Université de Nantes, Nantes, France; 8 CHU Nantes, 44093 Nantes, France

**Keywords:** Experimental models of disease, Preclinical research

## Abstract

Drug resistance limits the therapeutic efficacy in cancers and leads to tumor recurrence through ill-defined mechanisms. Glioblastoma (GBM) are the deadliest brain tumors in adults. GBM, at diagnosis or after treatment, are resistant to temozolomide (TMZ), the standard chemotherapy. To better understand the acquisition of this resistance, we performed a longitudinal study, using a combination of mathematical models, RNA sequencing, single cell analyses, functional and drug assays in a human glioma cell line (U251). After an initial response characterized by cell death induction, cells entered a transient state defined by slow growth, a distinct morphology and a shift of metabolism. Specific genes expression associated to this population revealed chromatin remodeling. Indeed, the histone deacetylase inhibitor trichostatin (TSA), specifically eliminated this population and thus prevented the appearance of fast growing TMZ-resistant cells. In conclusion, we have identified in glioblastoma a population with tolerant-like features, which could constitute a therapeutic target.

## Introduction

Glioblastoma (GBM) is the major and deadliest form of brain cancers in adult. Temozolomide (TMZ) is the standard of care for chemotherapy in patients with GBM. The resistance to this drug is modulated by DNA repair systems and in particular by the expression of O^6^-methylguanine-DNA methyl transferase (MGMT)^1,2^. The expression of MGMT is silenced by promoter methylation in approximately half of GBM tumors, and clinical studies have shown that elevated MGMT protein levels or lack of MGMT promoter methylation is associated with TMZ resistance in GBM^3,4^. However, almost invariably GBM recur even after an aggressive TMZ/irradiation regimen and recurrent tumors are highly resistant to treatments and often express MGMT even if absent in the original tumor^5^.

Resistance can however occur through multiple pathways that may be found independently or simultaneously^5,6^. Indeed the evolution of tumor cells under therapy can be viewed as a Darwinian process with replacement of sensitive clones by resistant clones^7^. This model is supported by the contention that tumors are composed of a large number of clones and that treatment could change the normal course of cancer evolution as dominant clones at diagnosis could be replaced by others, present within the cell population, because of the selective pressure of therapy^8,9^. Alternatively, the cancer stem cell hypothesis postulates a hierarchical organization of tumors, in which only a proportion of cells is tumorigenic and exhibits intrinsic resistance to most treatments^10^. Both models can account for tumor resistance and heterogeneity. Specific mutations have been shown in some cancers to be the major drivers of tumor resistance and growth^11^. Yet, specific inhibitors targeting these mutations almost always showed short-term success but did not preclude the development of resistance independent of the primary mutation. This is probably linked to the fact that differential drug responses can be observed even between cells that are genetically and epigenetically related^12^.

Drug resistance to treatments in cancer cells can thus either be intrinsic or adaptive and are governed by many mechanisms. Recently, persisters/tolerant cells, which were first observed in microorganism resistance to antibiotics, have been identified in tumors^13–17^. These cells have been shown, in lung cancer and melanoma cell lines, to precede and accompany resistance to tyrosine kinase inhibitors (TKI)^14–16^. However, little information on the role of tolerant populations in response to other drugs such as DNA-damaging agents is available.

We then studied, in vitro, in vivo, and in silico, the development of resistance to TMZ in a glioma cell line using a combination of phenotypic, metabolic, genomic, and single cell analyses. We identified an intermediate cell population crucial to the acquisition of resistance to the drug similar to tolerant/persisters population. We show that histone deacetylase inhibitors (HDI), eliminate specifically this population and prevent resistance to TMZ.

## Materials and methods

### Reagents

Temozolomide (TMZ) was from Interchim (Montluçon, France), all other drugs were from Sigma (Saint Louis, MO) unless otherwise noted. All cell culture products were obtained from Life Technologies (Carlsbad, CA).

### Cell culture

U251 and derivatives, A172 and LN18 (human glioblastoma cell lines) were cultured in DMEM (4.5 g/L glucose) enriched with 10% FCS (except LN18 in 5% FCS). U87 cells were cultured in DMEM (1 g/L glucose) supplemented with 10% FCS. All media contained 100 U/ml penicillin, 100 µg/mL streptomycin and 2 mM L-glutamine. Cells were maintained in 5% CO_2_ at 37 °C. U251 cell line authentication was certified by Eurofins Genomics (Ebersberg, Germany). All cell lines were routinely tested mycoplasma-free.

### Cytotoxicity assay and cell counts

MTT assays were performed as previously described^18^. Viable cell counts were performed using the Countess optics and image automated cell counter (Invitrogen, CA), after Trypan blue staining. Data are presented as percentage of viable cells after treatment compared to untreated cells (*N* = 3).

### Cell cycle analyses

After treatment for 3, 6, 9, 12, and 16 days with TMZ 50 µM, cells were harvested and fixed in 70% ethanol. After further wash with PBS, cells were stained with DAPI (Solution 3, Chemometec, Denmark) and quantified for their DNA content with the NucleoCounter® NC-250^TM^ system according to the protocol (*N* = 3).

### Clonogenic assay

U251 cells, pretreated or not with 50 µM TMZ at indicated times, were seeded at appropriate densities in 6-well plates. At least two dilutions of cells were used for each TMZ treatment time. Cultures were incubated for 1–2 weeks for colony formation. Then cells were stained with 0.5% crystal violet and colonies were counted using ImageJ (NIH). Colony forming efficiency of TMZ-treated cells was calculated as: [(number of clones/number of plated cells)/(number of wells) × plated efficiency for untreated U251^S^ cells] (*N* = 3).

### Metabolic studies

Mitochondrial oxygen consumption (OCR) and extracellular acidification rate (ECAR) were measured in non-buffered medium (NBM) supplemented with glucose (5 mmol/L), pyruvate (1 mmol/L), and glutamine (2 mmol/L) (NBMc) using a Seahorse XFp Analyzer (Agilent, CA) and the 8 wells cell culture miniplate (Agilent, CA). Briefly control and TMZ-treated cells were trypsined and 15,000 cells were plated/well. One well was kept free of cells for medium control. Next day, medium was changed for NBMc and after 1 h equilibration OCR and ECAR were measured three times over 15 min periods. Mean of these measures were then used for each sample (*N* = 3 with 2 or 3 technical replicates for each time point).

### Gene knockdown using siRNA

ON-TARGETplus—SMARTpool Human siRNA (CHI3L1: # L-012568-01, KLK5: #L-005916-00, #HB-EGF: L-019624, and FAT2: # L-011270-00, Dharmacon, CO) were transfected at final concentration of 15 nM in U251 cells with Lipofectamine RNAi Max (Life technologies) according to the recommended protocol. siRNA Scramble (Scr) (sc-37007, Santa-Cruz, TX) was used as negative control. Cells were reverse transfected when they were plated (day 1). A second classical transfection was done on day 4. Cells were treated with TMZ 50 µM on day 0 and day 3, and harvested at day 7 (*N* = 3).

### RNA extraction

For the gene expression assay and the RNASeq study, RNA extraction was performed as previously described^18^.

### Gene expression assay

RNA reverse transcription was performed using Maxima First Strand cDNA Synthesis Kit for RT-qPCR (Thermo Scientific, MA). Quantitative real-time PCR (qPCR) assays were performed in triplicates using the Perfecta™ SYBR® Green FastMix™, Low ROX™ (Quanta BioSciences, CA) and the real-time thermal cycler qTower (Analytik Jena AG, Germany). Fold change was calculated as previously described^18,19^ using RPLPO, TATA, and HGPRT as housekeeping genes (*N* = 5). One experiment was removed for CHI3L1 as two time points were missing.

### RNAseq

**Fig. 1:**
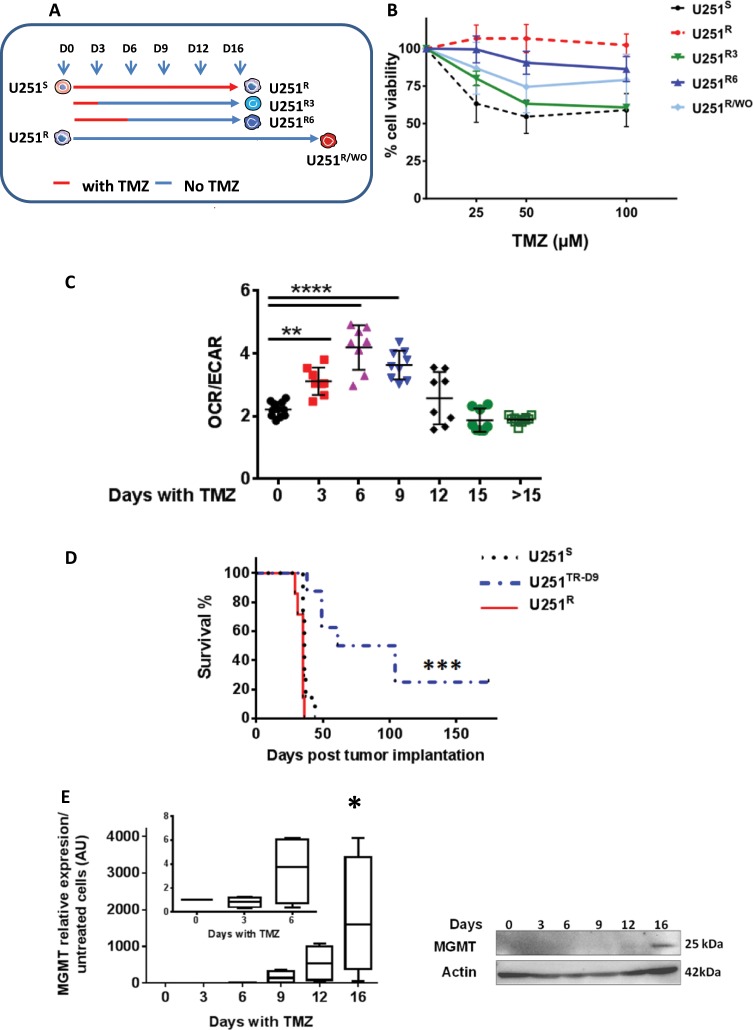
U251 cells follow a reproducible and distinctive pathway after TMZ treatment. **a** Experimental design of TMZ treatment to obtain different cell lines. U251^R3^, U251^R6^: cells treated respectively for 3 and 6 days with 50 µM TMZ and collected at day 16; U251^R/WO^: cells treated with TMZ for 16 days and then left untreated for 12 weeks. **b** Cell viability determined using MTT after 72 h exposure to TMZ. **c** Ratio of oxygen consumption rate (OCR) and extracellular acidification rate (ECAR) for U251^S^, and U251 cells treated with TMZ 50 µM (3, 6, 9, 12, and 15 days) measured by Seahorse Technology. Data are from three independent experiments with at least two replicates for each time point. **d** Kaplan–Meier survival curves. *N* = 7 for U251^S^, and U251^R^ and *N* = 8 for U251^TR-D9^. **e** MGMT expression in U251 over the time course of resistance acquisition, qPCR (left), protein analysis (right) with actin used as loading control.

Four resistant lines were obtained according to the protocol described in Fig. 1a. Library construction was performed for each time point with 500 ng total RNA with SureSelect Strand-Specific RNA Library Prep for Illumina Multiplexed kit (5190–6410, Agilent Technologies) according to Agilent_PrepLib_G9691-90010_juillet 2015_vD protocol. Purifications were carried out using NucleoMag NGS Clean-up and Size Select (Macherey-Nagel). Fragment size of libraries was controlled on a D1000 ScreenTape with 2200 TapeStation system (Agilent Technologies). Libraries with P5–P7 adapters were specifically quantified on LightCycler ® 480 Instrument II (Roche Life Science) and normalized with DNA Standards (1–6) (# KK4903, KAPABIOSYSTEMS-CliniSciences). Twelve picomolar of each library was pooled and prepared according to denaturing and diluting libraries protocol for the Hiseq and GAIIx (part#15050107 v02, Illumina) for cluster generation on the cBotTM system. Paired-end sequencing (2 × 100 cycles) was carried out in four lanes on HiSeq® 2500 system (Illumina) in TruSeq v3 chemistry according to the instructions of HiSeq® 2500 System Guide (part#15035786 v01, Illumina). After demultiplexing and quality control with fastQC_0.11.2 (http://www.bioinformatics. babraham.ac.uk/projects/fastqc/), Illumina adapter were trimmed with Cutadapt-1.2.1^20^ and reads with Phred quality score below 30 were filtered with prinseq-lite-0.20.3^21,22^. Reads were aligned against human hg19 reference genome with Tophat2.0.10^23^ counted with HTseq-count from HTSeq-0.5.4p5^24^ and differential analysis was performed with DESeq2^25^. The library obtained with one replicate D4 did not meet the quality check (low concentration) and was therefore eliminated from the bioinformatic analysis.

### Variant detection

The analysis was performed with GATK (v3.6.0) HaplotypeCaller from alignment files using hg19 reference genome. Variants were annotated and their predicted effects on known genes were calculated with SnpEff 4.3. Variants were then filtered out based on criteria: less than three replicates of D0 with the variant; DP > 10 in at least one replicate. Only variants with high putative impact were retained (Supplementary Info 1)^26,27^.

### Single-cell qRT-PCR

Gene expression analysis in single cells was measured using the C1 Single-Cell Auto Prep System coupled with the real-time PCR reader BiomarkHD (Fluidigm, CA) according to Fluidigm recommendations in untreated cells and in cells treated with 50 µM TMZ for 4, 9, 12, and 16 days. Single-Cell PreAmp IFC 10–17 and 17–25 µM were used, respectively for U251^S^, U251^R^, and U251^TR(D4, D9, D12)^. Results were analyzed using the Singular Analysis Toolset Software provided by Fluidigm. The list of primers is given in Supplementary Table 1.

### Western blot analysis

Proteins were extracted from adherent cells and separated as previously described^18^. Primary antibodies were as following: MGMT clone MT3.1 (#NB 100–692 Novus, MO) and anti-actin (clone C4, MAB1501 Millipore). Peroxydase-conjugated secondary antibodies (Jackson Immunoresearch Laboratories, PA) were detected using the ECL detection system (Amersham Biosciences, NJ) (*N* = 3).

### Elisa

CHI3L1 and HB-EGF enzyme like immunosorbent assay were performed using DuoSet Elisa kit (R&D, MN), according to the recommendations of the provider, in duplicate for each point. CHI3L1 was measured in the cells supernatants kept frozen to −80 °C until Elisa set up. For HB-EGF, ELISA was performed on whole cell lysate as previously described^28^ (*N* = 3).

### Mice and ethics statement

Male NSG mice (NOD.Cg-Prkdcscid Il2rgtm1 Wjl/SzJ) were purchased from Charles River Laboratories (France). They were bred in the animal facility of the University of Nantes (UTE, SFR F.Bonamy) under SPF status and used when aged between 6–12 weeks. The mice were housed on a 12 h light/darkcycle with access to food and water ad libitum. All procedures related to animal care and treatment were applied according to the European guidelines (Directive 2010/63/EU). The regional ethics committee for animal experiment, CEEA Pays de la Loire, (CEEA-PdL, France) provided agreement for the described study (Agreement #00186.02).

### Orthotopic injections of cells in NSG mice

Orthotopic injections of cells (10^4^ in 2 µL PBS), were performed using a stereotactic frame (Stoelting) at 2 mm on the right of the medial suture and 0.5 mm in front of the bregma, depth: 2.5 mm. U251^S^, U251^TR-D9^ (treatment in vitro for 9 days with 50 µM TMZ, +/−TSA 250 nM) and U251^R^ were injected on the same day. The experiments were unblinded without randomization. Animals were observed daily and euthanized when characteristic symptoms occurred, such as reduced mobility and significant weight loss (10%). At least four mice were used in each group (see also figure legends). Tumors were snap-frozen. Some tumors were analyzed and sections (2 µm) were used for histological staining by hematoxylin-eosine and immunohistochemistry with the following antibodies: rabbit anti-human anti-MHC class I Ab (clone EPR1394Y; Abgent, CA), mouse anti-human vimentin (clone 2A52, Novus bio), and mouse anti-human Ki67 (clone B56, Pharmingen BD, NJ). Slides were scanned using nanoZoomer 2.0 HT (Hamamatsu Photonics K.K., Hamamatsu, Japan). Images panels were prepared using Fiji and Image J.

### Public database analysis

cBioportal Genomics was used to analyze the expression of the four genes signature in The Cancer Genome Atlas (TCGA 2013). (http://www.cbioportal.org).

### Statistical analysis

All experiments were done at least in three biological replicates. Individual data points are plotted with mean ± SD. Statistical analyses were done using two-way Anova-multiple comparisons with difference considered statistically significant at *p* < 0.05 (GraphPad Prism 6). For survival curves comparison Log-rank test (Mantel-Cox) was used.

## Results

### Timeline of the acquisition of resistance to temozolomide by the U251 cells

U251 cells were treated every 3 days for up to 3 weeks with 50 μM TMZ (Fig. 1a), a dose relevant to the clinical condition^18^. A maximum of cell death (i.e., measured by caspase activity) was observed after 3 days of exposure to TMZ as described previously^18^. Cell cycle arrest (G2/M blockade) was observed between day 3 (D3) and D9 before U251 resumed their cell cycle G0/G1 (Supplementary Fig. 1). Morphologically (Supplementary Fig. 2), U251 cells exhibited a reproducible pattern of adaptation to the drug: in a first phase, cells exhibited a flattened cell body with few cellular extensions (D6–D9); in a second phase cells started to grow as colonies (D9–D12); in a third phase (i.e., after D12) cells recolonized the dish (Supplementary Fig. 2). The resistance to TMZ, measured by cell viability, was monitored on cells obtained after 16 days with or without treatment (Fig. 1b). We named U251^S^ (S for sensitive), the untreated cells and U251^R^ (R for resistant) cells treated with 50 μM TMZ for 16 days. As shown in Fig. 1b, the two cell lines exhibited different sensitivities to TMZ concentration up to 100 μM. U251 treated for 3 or 6 days with TMZ were then left untreated until D16 to generate U251^R3^ and U251^R6^, respectively. The resistance of these cells to TMZ was intermediate to that observed in U251^S^ and U251^R^ (Fig. 1b). On the other hand, U251^R^ left untreated for 3 months (U251^R^ wash out, U251^R/WO^) remained resistant to TMZ although to a lesser degree (Fig. 1b). However, after a brief exposure to 50 µM TMZ, U251^R/WO^ cells were again fully resistant. We analyzed the clonogenic survival of U251 cells during the course of TMZ treatment. As shown in Supplementary Fig. 3A, U251 cells treated with TMZ between D3 and D6 present a very low colony forming efficiency and U251^R^ (D16) exhibited limited clonogenicity compared to U251^S^ (D0).

Mitochondria and especially oxidative phosphorylation has recently been linked to treatment resistance in several cancers^29,30^. We thus analyzed the mitochondrial metabolism during the TMZ treatment using the Seahorse technology as previously described^31^. U251^S^ and U251^R^ cells have a comparable glycolytic metabolism while during the acquisition of resistance the ratio OCR/ECAR increased (Fig. 1c) due from day 6 to day 12 to the predominance of oxidative phosphorylation over glycolysis (Supplementary Fig. 3B, C).

Thus, TMZ-treated cells that transit from D3 to D12 have distinct metabolic properties, proliferation rates and morphologies and we called these cells U251^TR^ (TR for transient state). To evaluate and compare the tumorigenic properties of U251^S^, U251^TR^, and U251^R^ as well as the influence of the microenvironment, we implanted these cells in an orthotopic NSG mice model^32^. The survival of mice injected with U251^TR-D9^ cells (cells treated 9 days with TMZ 50 µM in vitro) was significantly increased over mice injected with U251^S^ or U251^R^ (Fig. 1d). The gross morphological aspects of the tumors derived from these cells did not show major difference at the time of death of the mice (Supplementary Fig. 4).

### Mathematical modeling of the acquisition of resistance to TMZ in U251 cells

The expression of MGMT has been associated with resistance to TMZ in GBM^33^ and in particular in U251 cells^18^. Therefore we measured MGMT in our cell line during the time of treatment by TMZ. MGMT was not present in U251^S^ cells but MGMT RNAs were significantly present after 9 days of exposure to TMZ and this expression increased continuously afterwards to reach a plateau at D16 (Fig. 1e left). At the protein level, the expression of MGMT was unambiguously detected only at D16 (Fig. 1e right). The resistance observed after D16 could be due to the selection of a preexisting clone or to an acquired program by some cells. We thus developed two different mathematical models corresponding to the two frameworks based on the proliferation properties of U251 cells with the expression of MGMT as the read out for resistance (see Supplementary Information 2).

**Fig. 2:**
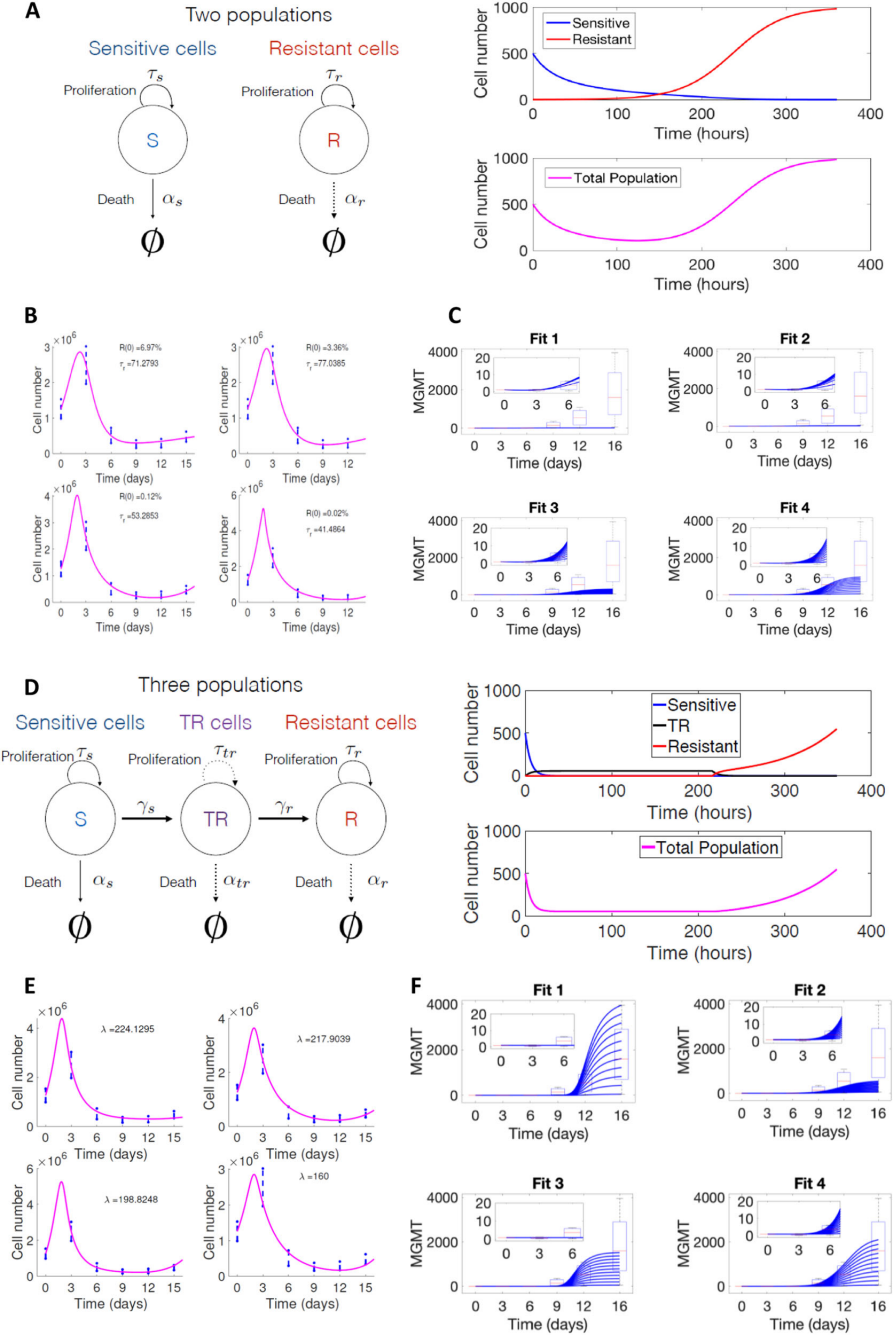
Mathematic model of resistance acquisition. **a** Scheme and basic dynamics of the clonal selection framework: (left) Schematic representation of the theory; (right) mathematical simulation of cell proliferation in the presence of TMZ (see Supporting information 2). **b** Data fittings according to the clonal selection interpretation. **c** MGMT variability within the clonal selection mode according to the 4 fits in b. **d** Scheme and basic dynamics of the acquired resistance model: (left) Schematic representation of the theory; (right) mathematical simulation of cell proliferation in the presence of TMZ (see Supporting information 2). **e** Data fittings according to the acquired resistance/transient population model. **f** MGMT variability within the acquired resistance model according to the 4 fits in e.

In the clonal selection model, a subpopulation with high levels of resistance is supposed to pre-exist in the original population (Fig. 2a) and will become the dominant clone upon the selection induced by TMZ. We used a system of two differential equations in combination with the standard least squares method to estimate the kinetic parameters, fitted to the experimental data. A large number of possible combinations for the initial fraction of resistant cells and the proliferation rate could fit the experimental data (Fig. 2b) and the initial proportion of resistant cells, i.e., U251^R^, would vary from 0.02 to 6.97% of the initial population. One parameter (*α*) was used to account for the MGMT expression ratio of resistant cells with respect to the basal values of the sensitive cells. In the early stages (first 6 days), the MGMT expression kinetics fits with the experimental results (insets in Fig. 1e). In contrast, at later times (days 12–16), it was not possible to fit the large variability observed in the MGMT expression (Fig. 2c), thereby indicating that a purely selection mechanism did not agree with our experimental results (see Supplementary Information 2).

According to the adaptive model, cells would evolve from a sensitive to a resistant phenotype through the acquisition of new expression of genes. Based on previous observations with TKI inhibitors^34^, this mechanism would not be direct, and will lead to a transient state, during which cells do not proliferate and do not undergo apoptosis. Thus, in addition to the sensitive and resistant populations, an intermediate population, similar to U251^TR^, was incorporated in the mathematical model (Fig. 2d). Our mathematical model incorporated the age-structure displayed by the nascent “TR” population (see Supplementary Information 2). Different parameter combinations were able to explain the cell numbers (Fig. 2e), now assuming a zero initial resistant population. Of note and in contrast with the previous model, using the MGMT expression, it was possible to find values and different parameter combinations that fit with all the experimental time points (Fig. 2f).

### RNA sequencing analyses of U251 cells following TMZ treatment

**Fig. 3:**
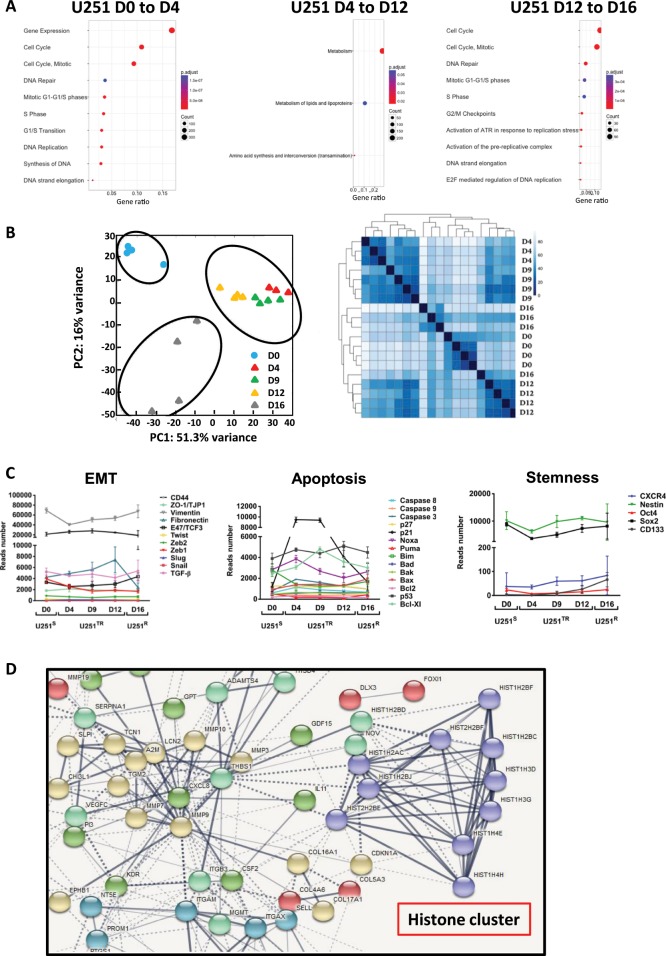
RNAseq analysis. **a** Reactome over-representation tests: pathways highlighted in three main evolutions during TMZ treatment of U251^S^. Gene ratio: proportion of mapped genes to each pathway. Count: number of genes. **b** Principal component analysis (PCA) (left), heatmap resulting from the transcriptome analysis of U251 after 0, 4, 9, 12, 16 days treatment with 50 µM TMZ (right). **c** Number of reads during acquisition of resistance for genes belonging to the apoptotic, EMT, and stemness families. **d** Functional protein association networks (STRING analysis) of genes over-expressed during the transient state (fold change > log2).

The distinct features observed in the U251^S^, U251^TR^, and U251^R^ and our mathematical models suggested a profound change in gene expression with TMZ treatment. We use RNA sequencing to validate these changes at D0, D4, D9, D12, and D16 after TMZ exposure. U251^S^ and U251^R^ did not appear significantly different regarding the pathways implicated in proliferation and DNA repair (i.e., activation of ATR). However, in the U251^TR^ most basal metabolisms were dysregulated (Fig. 3a). Principal component analysis (PCA) and hierarchical clustering analysis (HCA) of the normalized RNA sequencing data confirmed the three separate stages corresponding to U251^S^, U251^TR^, and U251^R^ (Fig. 3b). In addition, PCA indicated two groups, one containing the U251^TR^ population and the other one containing U251^S^ and U251^R^. Interestingly, U251^R^ exhibited a higher order of heterogeneity (Fig. 3b). Epithelial to mesenchymal-like transition (EMT), resistance to apoptosis and presence of cancer stem cells have been associated with treatment resistance in many cancers. We analyzed key genes implicated in EMT, apoptosis and stemness (Fig. 3c) in the different populations of the kinetic study. We found no differences between U251^S^, U251^TR^, and U251^R^ except for the expression of p21 in U251^TR^ (which could be related to cell cycle arrest) and fibronectin in U251^R^ (which could be related to cell migration/invasion). We used the STRING interactive network program to analyze pathways over-represented in U251^TR^ and we found one major molecular interaction network that centered on the histone pathway (Fig. 3d). We also analyzed the amount of mutations following the TMZ treatment and as shown in (Supplementary Fig. 5A) the mutation rates increased significantly between D0 and D4 and become stabilized thereafter. Of note, we did not identify any specific pathways with high mutation rates induced by the treatment.

### The U251^TR^ population induced by TMZ can be characterized by the expression of a limited series of genes

**Fig. 4:**
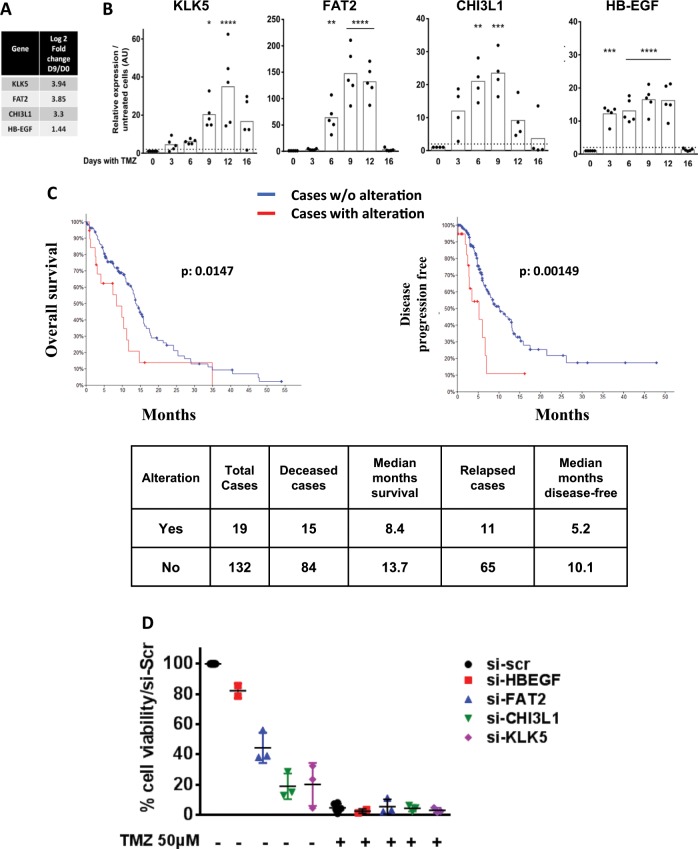
Markers of the transient phase. **a** Relative expression of the four selected markers at D9 in the RNAseq study. **b** Time course of KLK5, FAT2, CHI3L1 and HB-EGF by RT-qPCR in U251 cells treated with 50 µM TMZ every 3 days. Dotted line: fold change = 2. **c** TCGA glioblastoma database analysis for KLK5, FAT2, CHI3L1, and HB-EGF expression (RNAseq profile with a score threshold +/−2): Kaplan–Meier estimate of overall survival (left), and disease/progression-free (right). **d** Cell viability of U251 cells treated with siRNA directed against the respective genes and treated or not with TMZ 50 µM as detailed in Methods. Cells were collected and counted on day 7.

Based on RNA sequencing results (Fig. 4a), literature reports, our own previous data^28^, and qPCR validation, we selected four genes, KLK5, FAT2, CHI3L1 and HB-EGF, which were constantly and similarly over-expressed in U251^TR^. The transient expression of these 4 genes over the course of acquisition of resistance to TMZ is shown on Fig. 4b. HB-EGF and CHI3L1 had a similar kinetic at the protein level. (Supplementary Fig. 5B, C). We treated three other glioma cell lines having different MGMT status (U87, LN18, and A172 cells) with TMZ at various concentrations (250, 350, and 50 µM, respectively) and analyzed the expression of the four genes. As shown in Supplementary Fig. 6A, these genes appeared to be also regulated by TMZ although to a lesser extent. More importantly, in GBM patients the overexpression of KLK5, FAT2, CHI3L1, and HB-EGF significantly affected the overall survival and disease-free progression (Fig. 4c).

We used siRNA against these four genes in U251 cells (Supplementary Fig. 6B). As shown in Fig. 4d, U251^S^ treated for 7 days with gene specific siRNA or scrambled (scr) RNA in the presence or the absence of TMZ indicated that KLK5, CHI3L1, FAT2 had an impact on cell survival but no specificity toward TMZ treatment. In contrast HB-EGF, upregulated after TMZ treatment as previously described^28^, was found to play no role in cell survival (Fig. 4d). These results showed that these genes affected U251 survival independently of TMZ, validating the U251^TR^ as a tolerant rather than a specific resistant stage.

### Single cell analyses show that the transient population U251^TR^ is not uniform

**Fig. 5:**
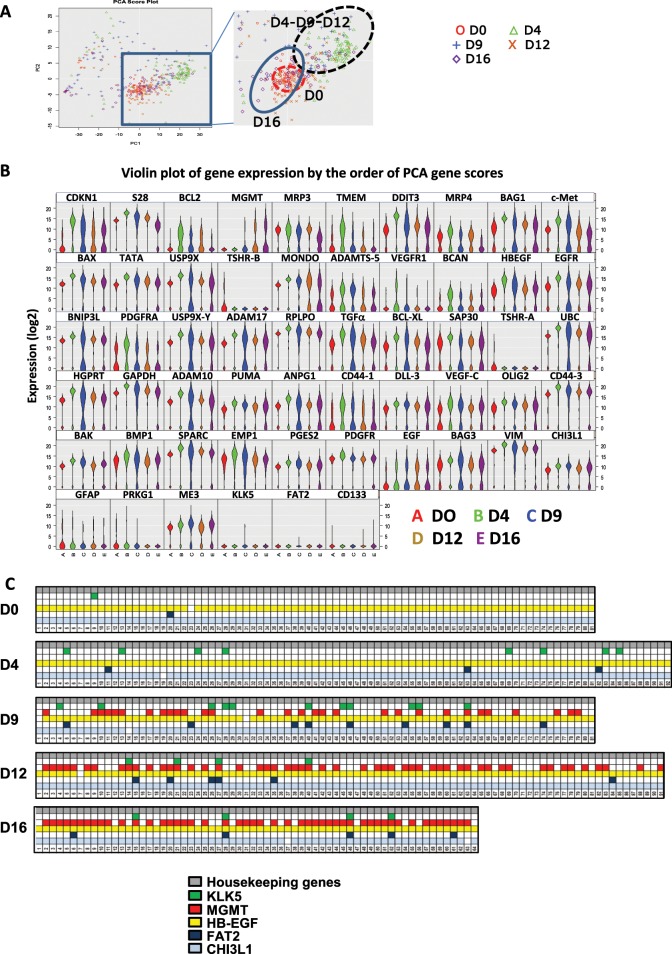
Single cell analyses. **a** PCA resulting from single cell analysis of untreated U251 cells, or U251 cells treated for 4, 9, 12, and 16 days with 50 µM TMZ. **b** Violin plot of gene expression. *Y*-axis represent (Threshold-Ct), meaning that the expression level is correlated to the y value. **c** Expression in U251 single cell of KLK5, FAT2, MGMT, HB-EGF and CHI3L1 over the course of treatment with 50 µM TMZ. Each square represents a cell and colors indicate that the expression of the gene was detected in the cell. All graphs are representative of one single cell analysis from three experiments.

Next, we analyzed the U251^TR^ population using a single cell approach based on the Fluidigm C1/HD Biomark quantitative PCR analyses. We analyzed 84 genes representative of different cellular functions including apoptosis, stemness along with the KLK5, FAT2 CHI3L1, and HB-EGF (see Supplementary Table 1) at D0, D4, D9, D12, and D16 after TMZ treatment. After unsupervised analysis, PCA representation allowed a distinction between U251^S^, U251^TR^, and U251^R^ (Fig. 5a). Violin plot representation (Fig. 5b) indicated that the distribution of most genes is unimodal during the TMZ treatment while a few genes appear to evolve from a unimodal to a bimodal distribution from U251^S^ to U251^TR^ and return to the unimodal stage in U251^R^ (i.e., Bcl-2, USP9X, EGFR, TGF α, ADAM10, ADAM17, and ANPG). Quite interestingly, most of these genes are associated with cell survival and/or were implicated in TMZ resistance in the literature. Next, we studied the expression cell by cell of KLK5, FAT2, CHI3L1, HB-EGF, and MGMT. The expression of MGMT appeared at D9, at first in a few cells and in most but not all cells after D12. On the other hand, the expressions of KLK5, FAT, CHI3L1 and HB-EGF were random among the cells at D4–D9. They were even co-expressed with MGMT in some cells at later stages (Fig. 5c). The expression of the four genes appeared to be independent. This suggested that the “TR” state does not necessarily correspond to a homogeneous population such as a single clone.

### Epigenetic targeting of U251^TR^ prevents the rise of U251^R^ in vitro and in vivo

**Fig. 6:**
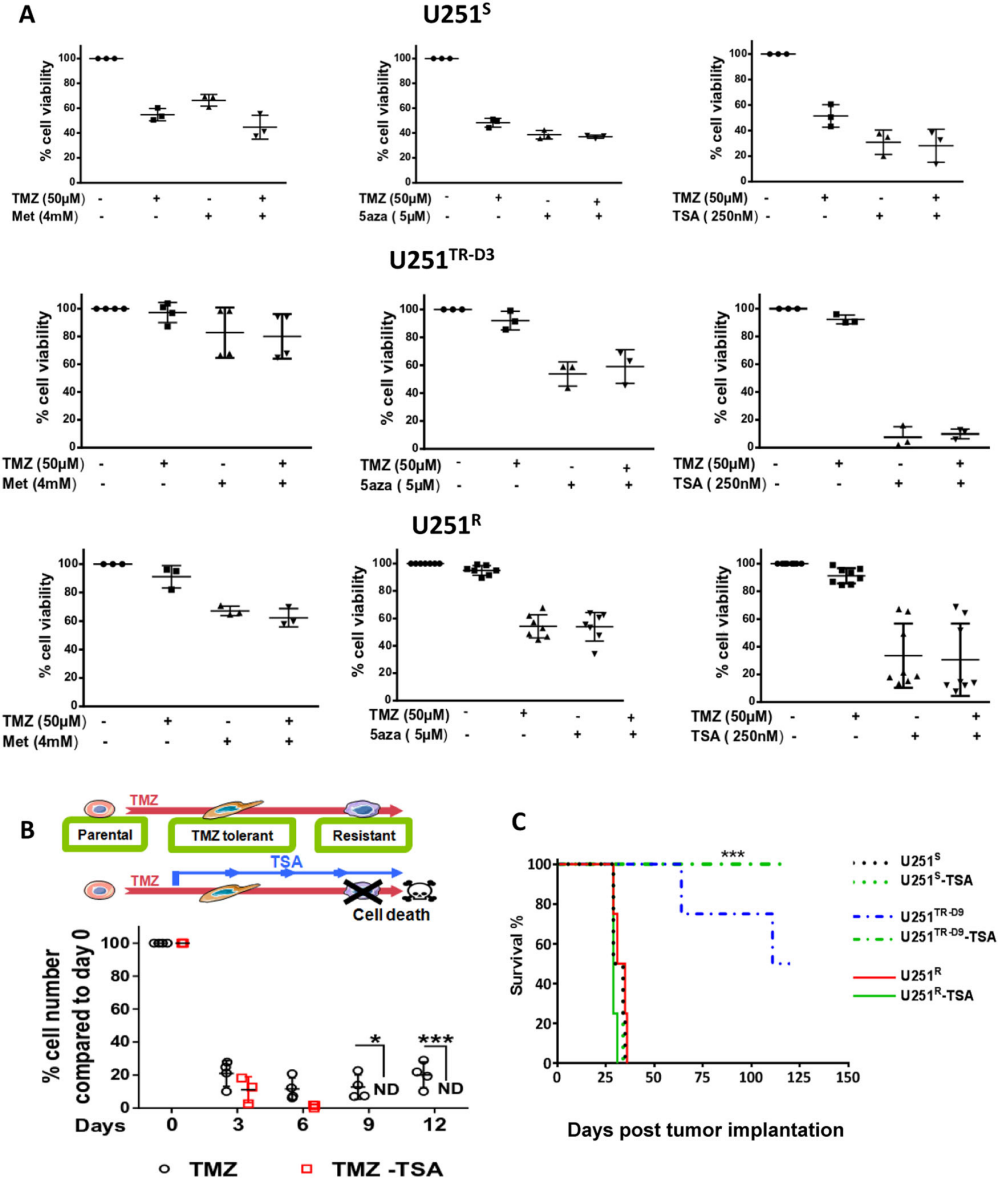
Drug sensitivity of U251^S^, U251^TR-D3^, and U251^R^ cells. **a** Cell viability after 72 h exposure of U251^S^, U251^TR-D3^, and U251^R^ to different drug combinations: (left) Metformin (4 mM), an inhibitor of mitochondrial respiration, (center) 5-aza (5 µM), a DNA demethylation agent and (right) TSA (250 nM), **a** histone deacetylase inhibitor (HDI). Data are expressed as percentage of cell viability compared to non-treated cells *N* ≥ 3. **b** Schematic representation of a therapeutic strategy to prevent emergence of resistant cells (top). Long-term effect of 250 nM TSA on U251^TR-D3^: cells were treated every 3 days with 50 µM TMZ in combination or not with 250 nM TSA (bottom). Cells were counted every 3 days. Cell number in U251^TR-D3^ was used as control. ND, not detectable. **c** Kaplan–Meier survival curves from mice injected orthotopically with U251^S^, U251^R^, and U251 ^TR-D9^ cells (±250 nM TSA) (*N* = 4 for each group).

Since U251^TR^ seemed to preferentially use mitochondrial oxidative-ATP-synthesis (Fig. 1c), we hypothesized that it could be more sensitive to Metformin (Met), a NADH dehydrogenase (complex I) inhibitor of mitochondrial respiration. Treatment with Met, in U251^S^, U251^TR^, and U251^R^ did not significantly affect the viability of U251^S^ or U251^TR^ cells, alone or in combination with TMZ but had a small effect on U251^R^ (Fig. 6a left). The expression of MGMT is related to the DNA methylation status of its promoter in glioma^35,36^. Thus, we treated the different U251 populations with the DNMT inhibitor 5-azacytidine, to decrease overall methylation. However, 5-Aza had no specific effect on U251^S^ and similar effects were seen in U251^R^ and U251^TR-D3^ (Fig. 6a center). As we had identified an upregulation of a histone cluster in the U251^TR^, Trichostatin A (TSA), an inhibitor of histone deacetylase (HDI) was tested on the different cell types. TSA had no significant effect on U251^S^, but specifically and significantly affected U251^TR-D3^ and to a lesser extent U251^R^ (Fig. 6a, right). To address the impact of a specific targeting of U251^TR^, we designed an experimental treatment in which U251 cells were treated by TMZ for 72 h (to generate U251^TR^) and then treated with TSA in combination with TMZ (Fig. 6b top). As shown in Fig. 6b (bottom), this treatment sequence efficiently eradicates U251^TR^ cells and prevents the onset of U251^R^ cells in vitro. The in vivo implantation of U251^S^, U251^TR-D9^, or U251^R^ cells treated or not with TSA confirmed that this treatment was efficient to reduce the growth of U251^TR-D9^ but did not affect that of U251^S^ and U251^R^ (Fig. 6c).

## Discussion

Recently, small populations of cancer cells, referred to as “cancer stem cells” or “drug tolerant cells”, have been identified in some cancers as being in a specific cellular state (slow cycling, low cell death, epigenetic alterations…) that allows them to survive drug treatment and to give rise of resistant and highly proliferative cancer cells^34^. Drug tolerant cells have been identified mainly in melanoma or lung cancer-derived cell lines treated with tyrosine kinase inhibitors (TKI) and identified as potential therapeutic targets to prevent resistance/relapse of cancers. In GBM, TKI induced a rapid and reversible state in cancer stem cells that was epigenetically regulated and associated with the expression of neurodevelopmental and quiescent signatures^37^.

We used a glioma cell line, as a model to explore the acquisition of resistance to TMZ, the current chemotherapy in GBM. This cell line did not express the DNA repair enzyme, MGMT. We found that resistance was accompanied by epigenetic changes with MGMT re-expression as a marker. Indeed, our previous results showed that promoting DNA methylation in GBM increased response to TMZ in association with the down regulation of different sets of genes including MGMT^38^. With the mathematical modelisation, although the clonal selection hypothesis could not completely be discarded when looking only at the cell number dynamics, if both the cell number curves and the MGMT expression results were considered, then the hypothesis that there was a transient state from sensitive to resistant cells appeared to provide a better underlying explanation of the experimental results.

Deep sequencing of control and TMZ-treated U251 cell-lines allowed us to identify new genes that are transiently over-expressed after TMZ treatment. Analysis of gene expression reveals that both qualitatively and quantitatively, the level of genomic heterogeneity appears to be reduced in treated cells during a transient period (i.e., D4–D12) before increasing at D16. A PCA based on RNAseq data reveal that sensitive cells (i.e., Day 0) form a cluster that is closer to resistant cells (i.e., day 16) than to the transient resistance state (Fig. 3b). From this study we selected four genes, which are implicated in cell survival and can be used as markers to define this state (Fig. 4). We performed, in control and TMZ-treated cells, single cell analyses of the expression of 88 genes including the four markers and others implicated in the cell death program and survival mechanisms (Fig. 5). This study showed that the transient state was not due to a subpopulation of cells but rather to a random distribution in the transient resistance state in the surviving cells. Analysis of the pathways over-expressed in U251^TR^ cells revealed that histones could undergo important modifications. Targeting of histone acetylation by TSA, indeed proved to efficiently eradicate these cells and thus to prevent the appearance of highly resistant and proliferative cells both in vitro and in vivo (Fig. 6).

Our results, coupling phenotypic analyses in cell death and proliferation rates to RNAseq techniques, show that the cell line upon TMZ treatment undergoes an initial rapid selection process. This step constitutes a “bottleneck” during which the cells rely on a few survival mechanisms and have a reduced heterogeneity. Next, the cells expand to become “fit and resistant” through many different mechanisms rendering these cells difficult to target. This hypothesis fits partially with the “drug-tolerant population” recently observed and described by several groups^13,15,16,34,39^ upon selection with TKI. However, few marked differences can be pointed out: our results are in favor of the acquisition of MGMT expression by a specific process during TMZ treatment rather than selection of a pre-existing clone^37,40,41^. The expression is not reversible when selective pressure by TMZ is removed, in contrast with other tumors, such as in non-small-cell lung carcinoma cells^16,42^ where reversibility to a former sensitive state was observed.

Taken together, our results show that the cells gradually acquire an irreversible resistance to TMZ and reveal a transient state with a reduced proliferation rate, G2/M arrest, distinctive mitochondrial metabolism and reduced aggressivity in vivo. The U251^TR^ population observed between D4 and D12 is not a uniform one as the four genes used as markers are not equally distributed along the cells. This result suggests that these genes are expressed in particular cells that cooperate together to create a transient state through their effect on cell survival. From this population and upon epigenetic remodeling the resistant population can thus emerge.

Our work provide a new rationale in the anti-GBM therapy with the use of HDI immediately after TMZ to ensure eradication of the tolerant population before the development of highly resistant fast growing tumors. This work has to be completed in primary cultures both in vitro and in vivo in mice.

## Supplementary information

Supplementary info 1

Supplementary Table 1

Supplementary Fig 1

Supplementary Fig 2

Supplementary Fig 3

Supplementary Fig 4

Supplementary info 2

Supplementary Fig 5

Supplementary Fig 6

## Data Availability

RNAseq data are available on NCBI trace Archive. PRJNA479416. www.ncbi.nlm.nih.gov/bioproject/?term=prjna479416.
