# Supplementary material for: Identification of a transient state during the acquisition of temozolomide resistance in glioblastoma
Source: Cell Death Dis. 2020 Jan 6;11(1):19. doi: 10.1038/s41419-019-2200-2 (PMC6944699; doi:10.1038/s41419-019-2200-2)
Supplement: Supplementary file 1 — Supplementary info 1 [file 41419_2019_2200_MOESM1_ESM.docx]

**Supplementary Information 1**:

List of putative impacts taken into account for variants filtering with SnpEff:

chromosome_number_variation

exon_loss_variant

frameshift_variant

stop_gained

stop_lost

start_lost

splice_acceptor_variant

splice_donor_variant

rare_amino_acid_variant

missense_variant

disruptive_inframe_insertion

conservative_inframe_insertion

disruptive_inframe_deletion

conservative_inframe_deletion

5_prime_UTR_truncation+exon_loss_variant

3_prime_UTR_truncation+exon_loss

splice_branch_variant

splice_region_variant

stop_retained_variant

initiator_codon_variant
