# Supplementary material for: Identification of a transient state during the acquisition of temozolomide resistance in glioblastoma
Source: Cell Death Dis. 2020 Jan 6;11(1):19. doi: 10.1038/s41419-019-2200-2 (PMC6944699; doi:10.1038/s41419-019-2200-2)
Supplement: Supplementary file 2 — Supplementary Table 1 [file 41419_2019_2200_MOESM2_ESM.docx]

Supplementary table 1: genes used in single cell analysis

| **ADAM10** | **KRT13** | **CHI3L1** | **RPS4Y1** | **DKK1** | **BCL2** | **BAG3** | **MONDO** |
| --- | --- | --- | --- | --- | --- | --- | --- |
| **ANKRD1** | **ADAMTS 5** | **CCND2** | **DDX3Y** | **BMPER** | **TSHR-B** | **CRHR1** | **c-Met** |
| **GMR8** | **ADCY2** | **FAT2** | **BMPER** | **EFEMP** | **ADAM17** | **MGMT** | **EGF** |
| **ME3** | **CD137** | **SAP30** | **PRKG1** | **SPARC** | **PRKG1** | **USP9X** | **HGF-1** |
| **PGES2** | **COX2** | **IGF** | **TGFα** | **TMEM** | **KLK5** | **CDKN1A** | **ANPG1** |
| **BAX** | **BAK** | **MCL1** | **HBEGF** | **CD137** | **VEGFC** | **SKG3** | **BAG1** |
| **PUMA** | **BNIP3L** | **BCL-XL** | **PDGFRA** | **USP9X-Y** | **IEX-1** | **EFEMP-1** | **HER4** |
| **CD133** | **VIM** | **CADH** | **EMP1** | **FAT2** | **MRP3** | **MRP4** | **TSHR-A** |
| **VEGFR1** | **MGMT** | **LEMD1** | **DDIT3** | **CD44-1** | **CD44-3** | **BCAN** | **EGFR** |
| **PDGFR** | **OLIG2** | **DLL3** | **GFAP** | **MEDAG** | **SCN9A** |  |  |
| **TATA** | **RPLPO** | **GAPDH** | **UBIQUITIN** | **HGPRT** | **S28** |  |  |

**Housekeeping genes**

Primers sequences will be available on demand
