# Supplementary material for: Identification of a transient state during the acquisition of temozolomide resistance in glioblastoma
Source: Cell Death Dis. 2020 Jan 6;11(1):19. doi: 10.1038/s41419-019-2200-2 (PMC6944699; doi:10.1038/s41419-019-2200-2)
Supplement: Supplementary file 3 — Supplementary Fig 1 [file 41419_2019_2200_MOESM3_ESM.pdf]

A

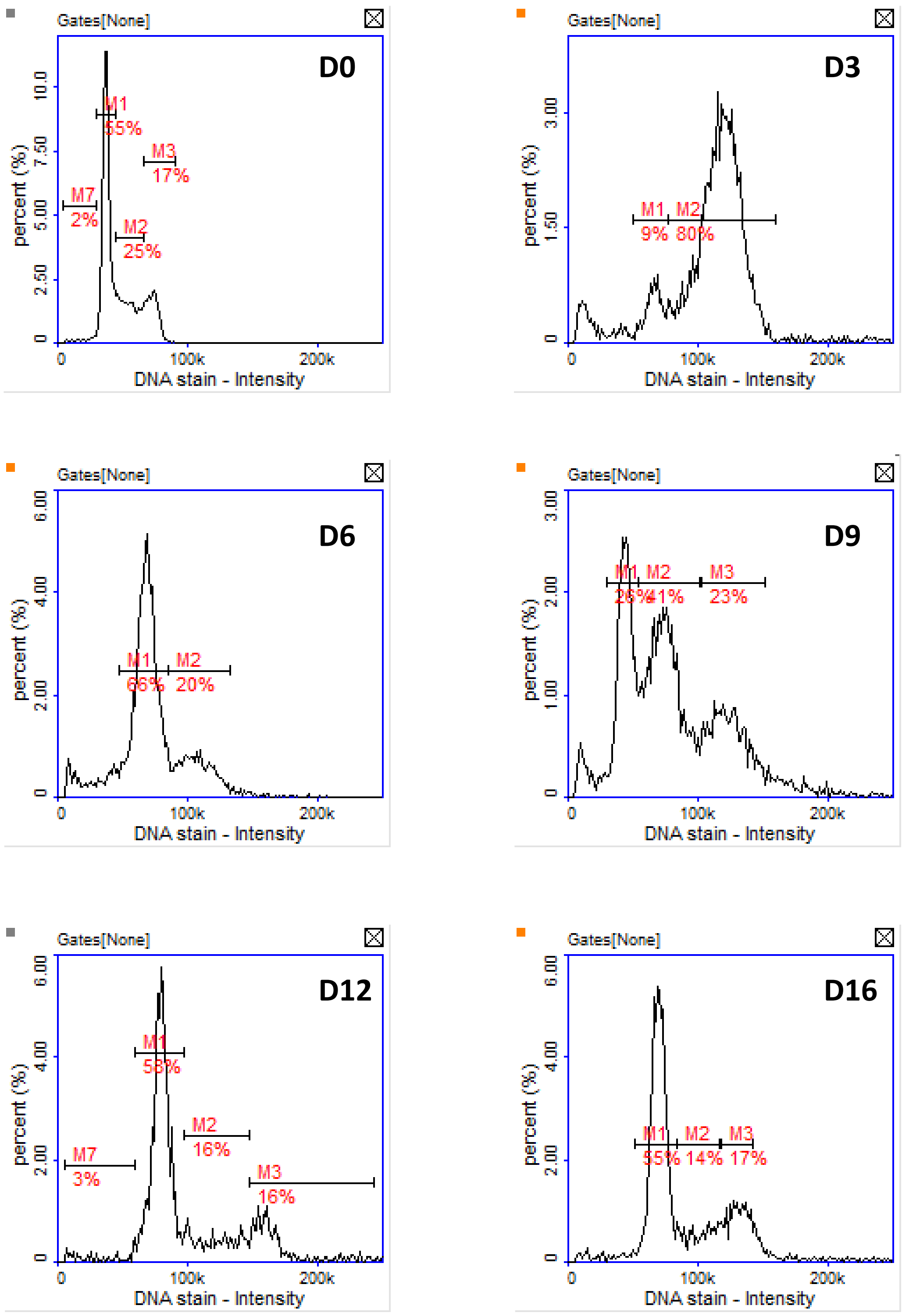

Figure S1. Cell cycle analysis of U251 cells without treatment and treated for 3, 6, 9, 12 and 16 days with TMZ 50µM. Data from one experiment representative of three.

M Rabe et al Supplementary Fig 1
