# Supplementary material for: Identification of a transient state during the acquisition of temozolomide resistance in glioblastoma
Source: Cell Death Dis. 2020 Jan 6;11(1):19. doi: 10.1038/s41419-019-2200-2 (PMC6944699; doi:10.1038/s41419-019-2200-2)
Supplement: Supplementary file 4 — Supplementary Fig 2 [file 41419_2019_2200_MOESM4_ESM.pdf]

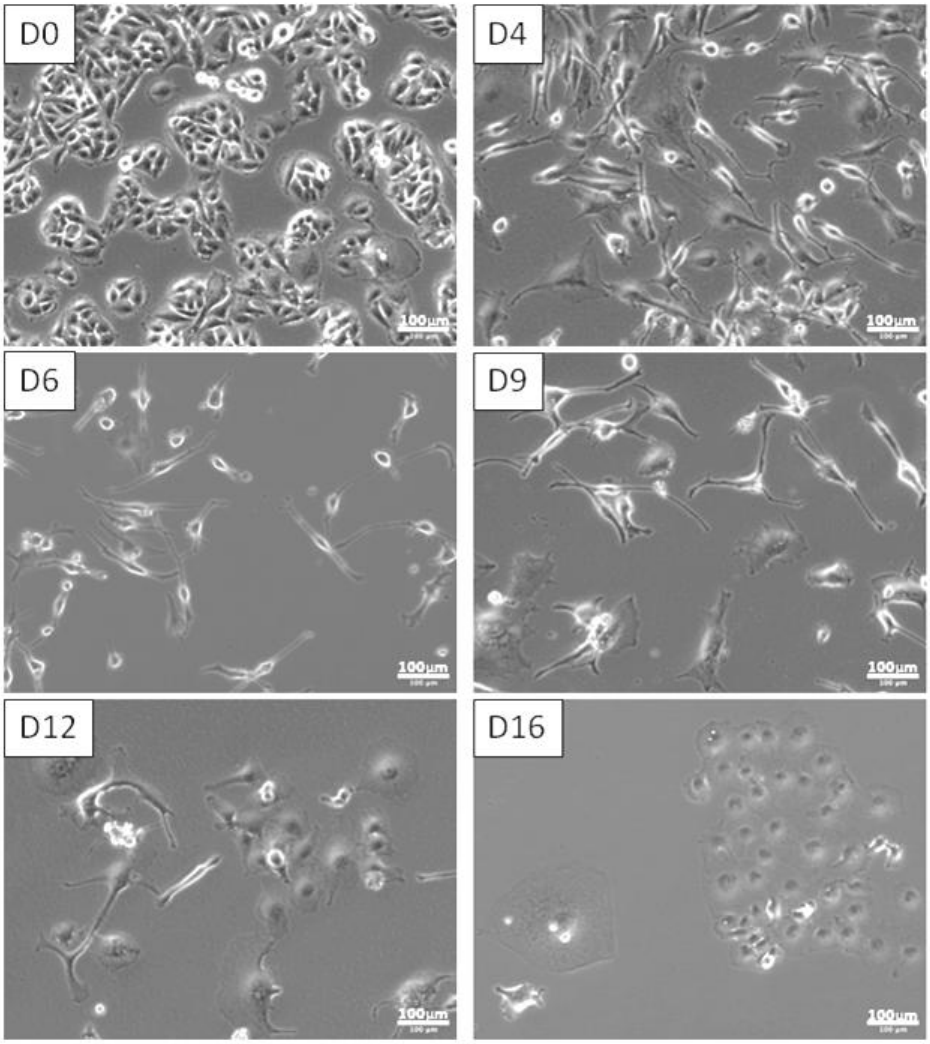

Figure S2. Morphological aspect (light microscopy, x 32) of U251 treated with 50µM TMZ every 3 days from day 0 (D0) to day 16 (D16). Scale 100µM
