# Supplementary material for: Identification of a transient state during the acquisition of temozolomide resistance in glioblastoma
Source: Cell Death Dis. 2020 Jan 6;11(1):19. doi: 10.1038/s41419-019-2200-2 (PMC6944699; doi:10.1038/s41419-019-2200-2)
Supplement: Supplementary file 5 — Supplementary Fig 3 [file 41419_2019_2200_MOESM5_ESM.pdf]

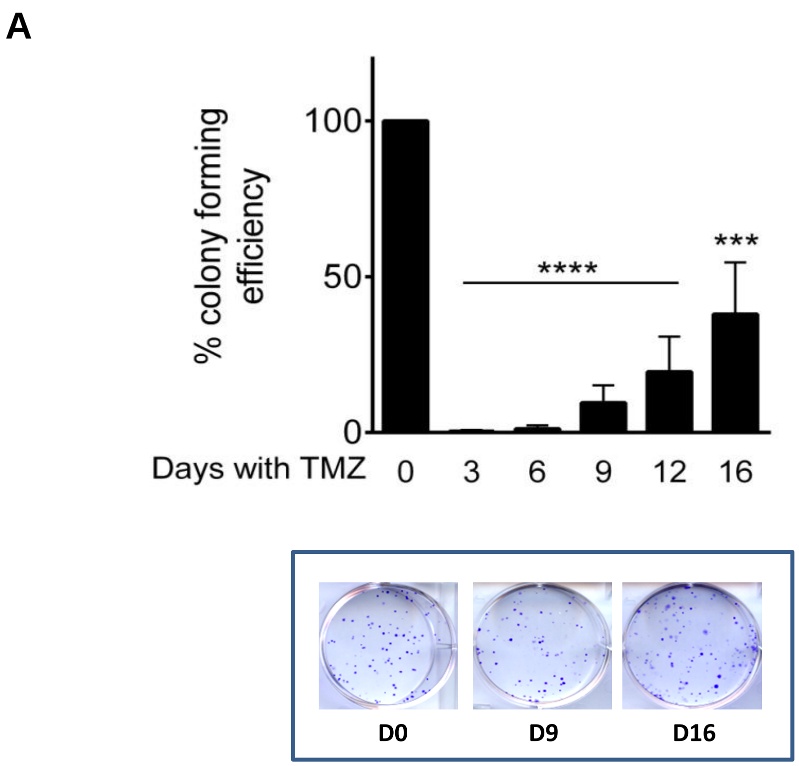

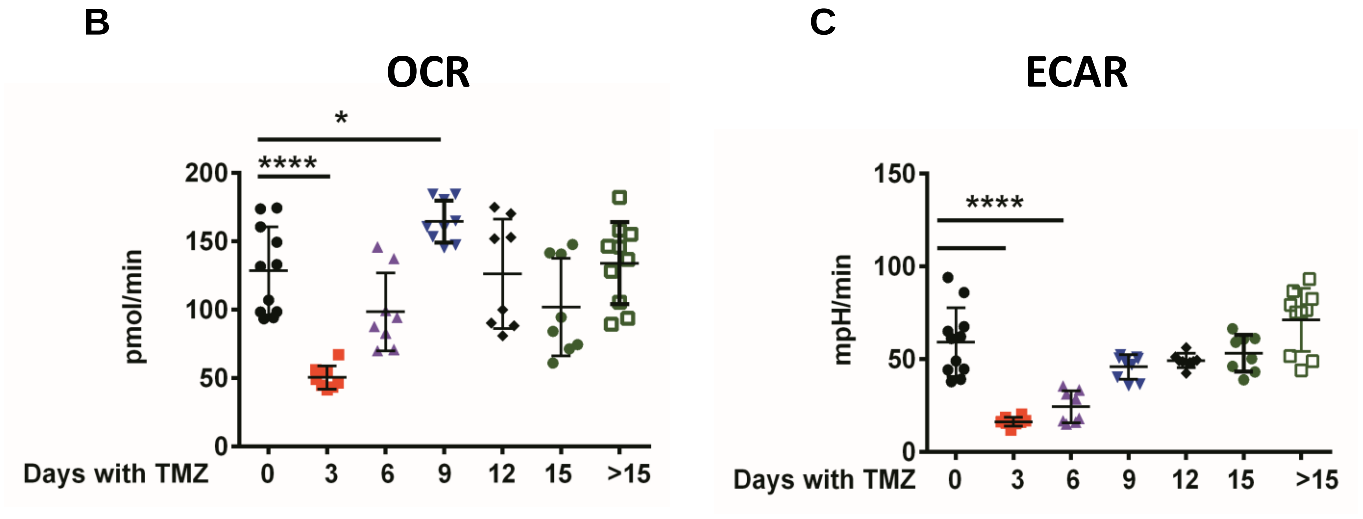

Figure S3. **(A)** Colony forming efficiency of U251 during the time course of treatment: U251 cells were exposed to TMZ 50µM for 0,3,6,9,12,or 16 days and then cells were respectively plated at 100, 30000, 15000, 2000, 500 and 300 cells per well.
(B-C) Profile of **(B)** OCR and **(C)** ECAR during acquisition of resistance measured with the Seahorse XFpAnalyser. Data are from three independent experiments with at least 2 replicates for each time point.
