# Supplementary figures and images for: Identification of a transient state during the acquisition of temozolomide resistance in glioblastoma
Source: Cell Death Dis. 2020 Jan 6;11(1):19. doi: 10.1038/s41419-019-2200-2 (PMC6944699; doi:10.1038/s41419-019-2200-2)

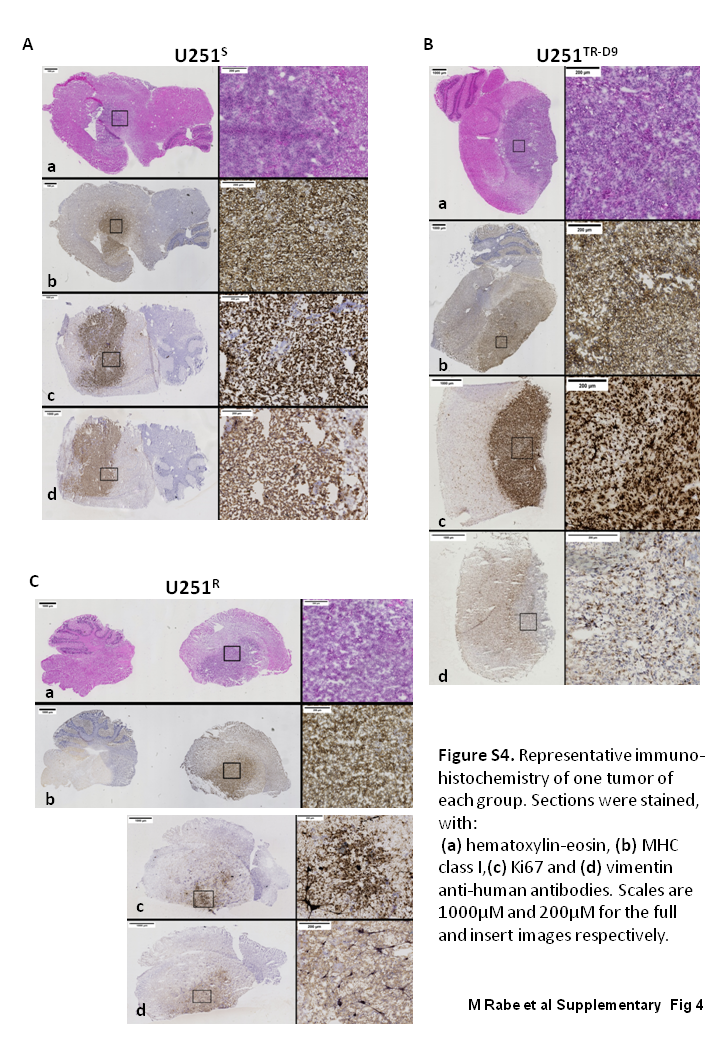

Supplement: Supplementary file 6 — Supplementary Fig 4 [file 41419_2019_2200_MOESM6_ESM.tif]
