# Supplementary material for: Identification of a transient state during the acquisition of temozolomide resistance in glioblastoma
Source: Cell Death Dis. 2020 Jan 6;11(1):19. doi: 10.1038/s41419-019-2200-2 (PMC6944699; doi:10.1038/s41419-019-2200-2)
Supplement: Supplementary file 7 — Supplementary info 2 [file 41419_2019_2200_MOESM7_ESM.docx]

**Supplementary Information 2: Mathematical models**

**Experimental results:**

**Fig. SI2.1: Evolution of U251 cell numbers sampled at days 3; 6; 9; 12 and 15. The solid blue line and the vertical error bars correspond to the mean and standard deviation of four replica experiments.**

This behaviour suggests that TMZ does not exhibit an immediate effect on cancer cells. To better understand the underlying processes, we looked at information in the existing literature and found the following information:

• In References [1]-[3] experiments with the same cell line, U251, were carried out in the absence of drugs. From the measured results for cell growth, we can "estimate" the doubling time τs of these cells. The obtained values were τs = 34.35; 28.86 and 34.75 hours, respectively. Therefore, the doubling time used in the *in silico* simulations should be within those values.

• In Ref. [4] changes in survival of U251 cells were measured with a concentration of 150 μM of TMZ. No significant changes in cell survival were observed during the first 24 h. See Ref. [4] Fig. 1.

• In another work (Ref. [5]), cell survival was measured after 48h in the same cell line with different concentrations of TMZ. In the experiments with 50 μM, which correspond to the same concentration employed in the present study, there was almost no significant difference in cell survival during the first two days. See Ref. [5] Fig. 2.A .

Therefore, very similar results during the initial course of TMZ administration on U251 cells have been previously obtained and support our experimental findings. Thus, in our mathematical model we make a first hypothesis: TMZ does not display an immediate effect on U251 cell survival for a concentration of 50 μM. The time delay required to observe a significant TMZ-induced cell death is around two days. We further assume that this change from no response to total response is progressive. Hence, a drug response function will be defined and will be present as a product factor in all those processes where TMZ is involved. This function depends on time, and goes from 0, corresponding to no effect, to 1, implying a total effect. The profile of this function is depicted in Fig. SI2.2.

**Fig. SI2.2: Effect of TMZ on U251 cells during drug administration, staring at time t = 0.**

**Mathematical analyses:**

Here we wished to address the following questions. Is the final resistant population due to a Darwinian-type selection of a resistant clone already present from the beginning? Or, else, does a resistant population emerge as a consequence of a Lamarckian-type of evolution leading to phenotypic variation from which the non-genetic inheritance of an acquired adaptive trait can be transmitted to the offspring? These two questions reflect two possible hypotheses. To identify which is more plausible, we develop two different mathematical models according to the two premises.

**1.1 Clonal Selection Model**

The idea behind this hypothesis is the existence of a subpopulation with high levels of resistance. The presence of the drug will result in an important decrease of the sensitive population, while the resistance cells will continue to grow eventually becoming the dominant clone (see Fig. 3a).

From a mathematical point of view, such dynamics can be easily described with a system of two ordinary differential equations. Assuming a logistic growth, the equations can be written as:


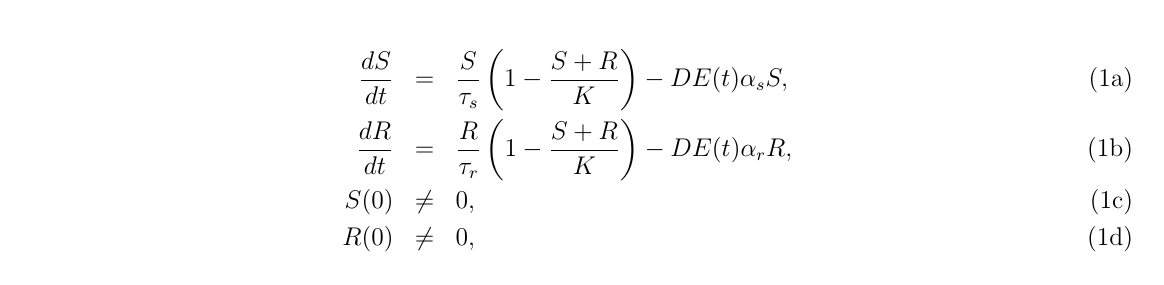


where DE(t) is the drug effect function mentioned before. With these equations and using a least

squares method to estimate the parameter values, it is possible to fit the experimental data. However,

due to the number of parameters to be evaluated there are several combinations that could explain the experimental results. In Fig. 3B we present four possible fits, with their corresponding parameters presented in Table 1.

**Table SI2.1: Estimated parameter values within the clonal selection interpretation. Parameter K was fixed before the analysis.**

**1.2 Acquired Genetic Expression**

Within the realm of the Lamarckian-type evolution, we can think of the emergence of resistant cells not due to a pre-existing clone but as a consequence of genetic noise amplified by the presence of the drug. In this framework we hypothesize that cells can evolve from a sensitive phenotype towards a resistant one, so cells which were initially sensitive can acquire partial or total resistance to the administered drug. This mechanism is not direct, and cells need to go through a transient state, in which they remain non-proliferative but significantly reduce their apoptosis induced by the drug. Therefore, in addition to the sensitive and resistant populations, we incorporate another population, called ”transitory resistant" (TR). The basic scheme is shown in Fig. 3d. To represent this new dynamic we put forward an age-structured model, including a new variable, the age *a* (time) of transition from TR to resistant cells. Since sensitive and resistant cells do not depend on this variable, it is not necessary to include it in both populations. Our system can be written as:


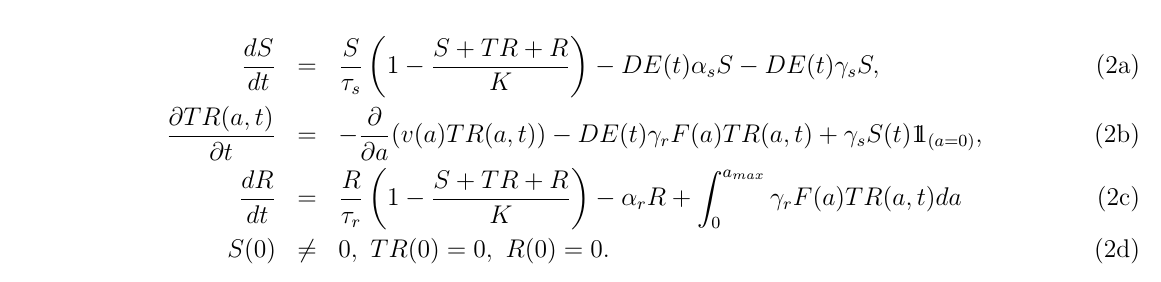


In these equations, F(a) denotes a distribution function. Fig. SI2.3 illustrates an example of this function.

 **Fig. SI2.3: Functions used in this simulations**

As in the clonal selection model, there are several combinations of parameters that explain the measured cell number at different times. In Fig. 3d, four examples of possible combinations are shown, with the corresponding parameters collected in Table SI2.2. An important feature of these simulations is that, initially, only sensitive cells are present in the cell cultures.

**Table SI2.2: Estimated parameter values for the acquired genetic expression model**.

**2. MGMT expression**

In this section, we analyze the MGMT expression of the total population, which is a biomarker of

resistance. During the first six days there is almost no change in MGMT expression, with an important

increase occurring from day 9. Changes in population's MGMT expression are shown in Fig. 2a,

where the dynamics of 4 different experiments are plotted, and represented with the corresponding boxplots.

**2.1 Clonal Selection Hypothesis**

Cancer cells have basal values of the protein MGMT, and its overexpression is related with a resistant

phenotype. Within the clonal selection hypothesis, the normalized MGMT expression of the total population can be easily modelled by means of the following equation:


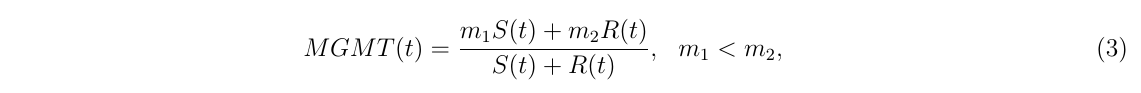


where *m_1_* and *m_2_* are weight parameters. To analyze how MGMT expression in cancer cells is changing with the treatment, our obtained results are compared with untreated cells, i.e., with the MGMT expression at day 0. Therefore, the relative MGMT expression is given by:


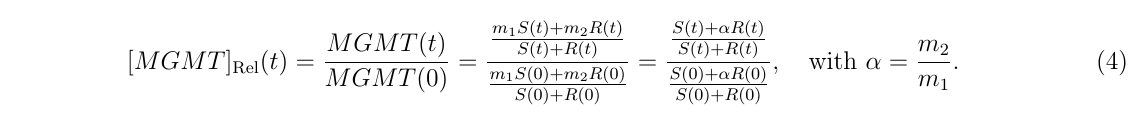


Note that due to the employed normalization, there is only one parameter, α, which stands for the MGMT expression ratio of resistant cells with respect to the basal values of sensitive cells (Table SI2.3). We wish to set forth two relevant questions. Can the MGMT changes observed in the experiments be explained with this theory? Is the final variability in agreement with the observations?

In Fig. 3c we show how different values of fits provide dissimilar MGMT curves. In Fit 3 and Fit 4 plots, with a low initial percentage of resistant cells (0.12 and 0.02% respectively), the clonal selection interpretation could explain some of the observed experimental results. However, even though the variability is well captured during the first six days; this mathematical model is not able to explain the high variability from day 9 to 16, as all the MGMT curves are well below the median.

**Table SI2.3: estimated parameter value for the clonal model**

**2.2 Acquired Resistance Hypothesis**

Within the acquired resistance hypothesis, and assuming TR cells are changing their MGMT expression from the basal value *m_1_* to the resistant level *m_2_* according to their age, MGMT expression can be expressed as follows:


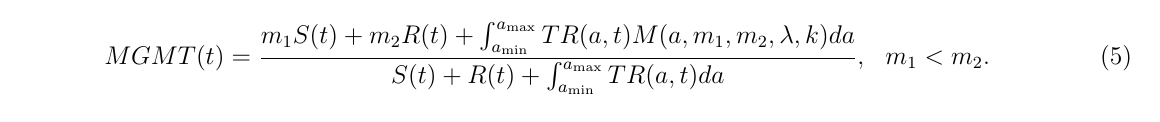


where,


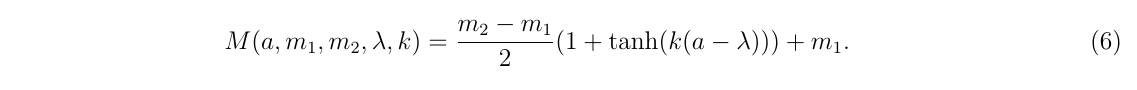


is a function ranging from m_1_ to m_2_, depending on the parameter $\lambda$ and *k*. The relative expression would depend also on the same parameter, $\alpha$


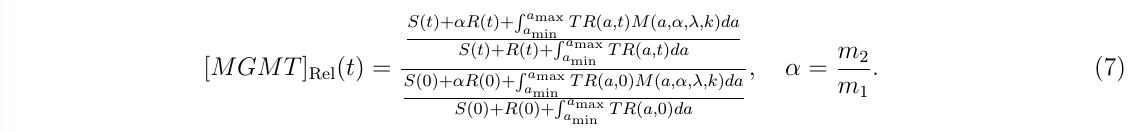


As in this hypothesis there are only sensitive cells at the beginning, *R(0)=0* and *TR(a,0)=0* $\forall a>0$, eq. (7) becomes


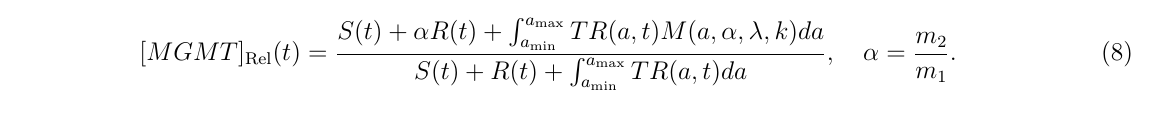


where,


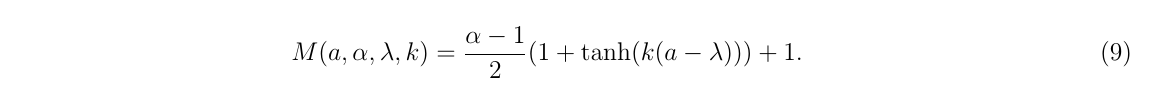


It is reasonable to think that cells are changing from TR to resistant cells as fast as they are changing their MGMT expression from *m_1_* to *m_2_*. Therefore, for each fit, those values are the same as those shown in Table 2. As mentioned before, there are many parameter values that could explain the cell number curves. Each combination of the different parameters provides dissimilar MGMT curves, where the parameters λ and *k* are very important in this analysis (Table SI2.4). To gain insight of the meaning of these parameters, in Fig. SI2.4, the influence of both parameters is analyzed. The value of λ marks the time at which cells have acquired half of the total MGMT, and the value of *k* provides information about how fast is the process of resistance acquisition; the larger the value of *k*, the faster the process becomes.

**Fig. SI2.4: example of the effect of λ and κ in the function**

Fig. 3C shows the different behaviours of the different fits, with their corresponding
α min and α max values shown in Table SI2.4. We analyze the corresponding Fig.s: There is almost no variability during the first 9 days in fits 1 and 3 plots. After that, and depending on the value of α, fit is possible to get high variability at day 16. With this theory and the present model, when *k* is around 0.75 it is possible to get a large variability during the first 9 days. In the fourth fit, different values of α provide high variability during all the experimental days, as observed in the experiments. Let's notice that with this theory, as only sensitive cells are present at the beginning, and only resistant cells at the end (or almost only), the final value of MGMT is close to α.

**
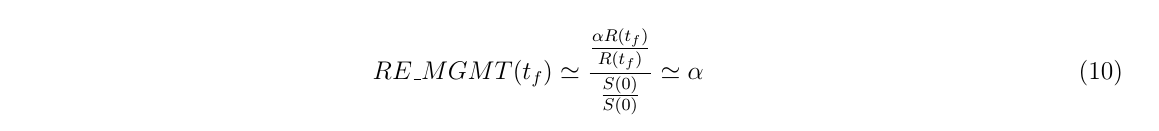
**

**Table SI2.4: estimated parameter for the acquired resistance model**

**3. Conclusions**

After making a number of hypothesis about the delay in the effect of TMZ on U251 cells, we have seen that both theories are able to explain the behaviour of the cell number dynamics. In both of them, there are several possible combinations for the parameters. The predictions of the mathematical models, representing each of the theoretical frameworks, for the cell number behaviour can be very similar and, thus, when fitting the current experimental results, it is not possible to decide with certainty which of the two interpretations, clonal selection versus acquired resistance, yields the best explanation. Now, if the MGMT expression of the population is followed over time then, after making the corresponding hypotheses, we saw how the MGMT expression changed according to the two different interpretations. In both of them, there was only one new parameter, α, representing the MGMT expression ratio of resistant cells with respect to the sensitive ones. Within the clonal selection framework, it was possible to find values of α that could explain some of the observed experimental results, those with the lower final values of MGMT. However, for all the explored parameter combinations it was no possible to find any α that could explain the high variability at day 16. Presumably, had the follow-up of the cell populations being extended further in time, the agreement would have worsened rather than improved. In contrast, within the acquired resistance hypothesis, it was possible to find values of α and different parameter combinations that could explain the variability during all the experimental time points. Therefore, although one cannot completely discard the clonal selection hypothesis when looking only at the cell number dynamics, we may conclude that if both the cell number curves and the MGMT expression results are considered, then the hypothesis that there is a transient state from sensitive to resistant cells appears to provide a better underlying explanation of the experimental results. It should be pointed out that it is not possible to rule out that a combination of the two interpretations may actually take place simultaneously. Since they are not mutually exclusive, it could thus occur that both a small initial population of resistant cells is present and also that sensitive cells may traverse an intermediate TR state before becoming fully resistant.

**4. References**

[1] Austin M. Guo, Ju Sheng, Gloria M. Scicli, Ali S. Arbab, Norman L. Lehman, Paul A. Edwards, John R. Falck, Richard J. Roman and A. Guillermo Scicli. Expression of CYP4A1 in U251 Human Glioma Cell Induces Hyperproliferative Phenotype in Vitro and Rapidly Growing Tumors in Vivo Journal of Pharmacology and Experimental Therapeutics October 1, 2008, 327 (1) 10-19.

[2] Liu, X., Li, G., Su, Z., Jiang, Z., Chen, L., Wang, J. ... Liu, Z. (2013). Poly(amido amine) is an ideal carrier of miR-7 for enhancing gene silencing effects on the EGFR pathway in U251 glioma cells. Oncology Reports, 29, 1387-1394.

[3] Wang, F., Sun, J., Zhu, Y., Liu, N., Wu, Y., & Yu, F. (2014). MicroRNA-181 inhibits glioma cell proliferation by targeting cyclin B1. Molecular Medicine Reports, 10, 2160-2164.

[4] Murphy, AC., Weyhenmeyer B, Schmid J, et al. Activation of executioner caspases is a predictor of progression-free survival in glioblastoma patients: a systems medicine approach. Cell Death Disease.2013; 4(5):e629.

[5] Cheng Y, Sk UH, Zhang Y, et al. Rational Incorporation of Selenium into Temozolomide Elicits Superior Antitumor Activity Associated with Both Apoptotic and Autophagic Cell Death. Wu GS, ed. PLoS ONE. 2012;7(4): e35104.
