# Supplementary material for: Identification of a transient state during the acquisition of temozolomide resistance in glioblastoma
Source: Cell Death Dis. 2020 Jan 6;11(1):19. doi: 10.1038/s41419-019-2200-2 (PMC6944699; doi:10.1038/s41419-019-2200-2)
Supplement: Supplementary file 8 — Supplementary Fig 5 [file 41419_2019_2200_MOESM8_ESM.pdf]

A

B

C

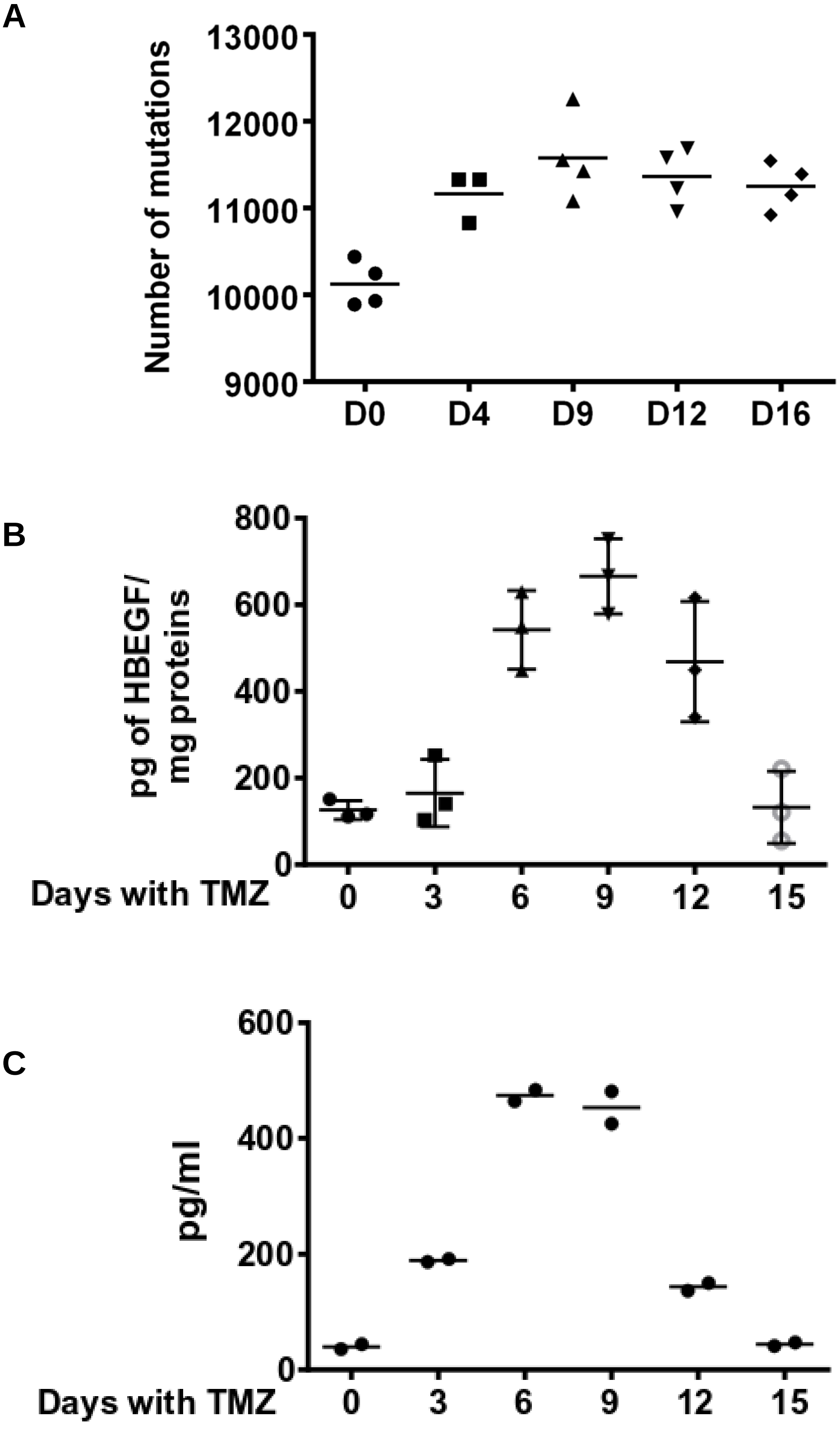

Figure S5. (A) Global mutations number during TMZ treatment, identified from RNA sequencing.

(B) HB-EGF in whole cell lysate during acquisition of resistance N=3

(C) CHI3L1 protein in the supernatant of U251 cells over the 15 days treatment. Graph is representative of one experiment of 3 (Elisa: n=2).
