# Supplementary material for: Identification of a transient state during the acquisition of temozolomide resistance in glioblastoma
Source: Cell Death Dis. 2020 Jan 6;11(1):19. doi: 10.1038/s41419-019-2200-2 (PMC6944699; doi:10.1038/s41419-019-2200-2)
Supplement: Supplementary file 9 — Supplementary Fig 6 [file 41419_2019_2200_MOESM9_ESM.pdf]

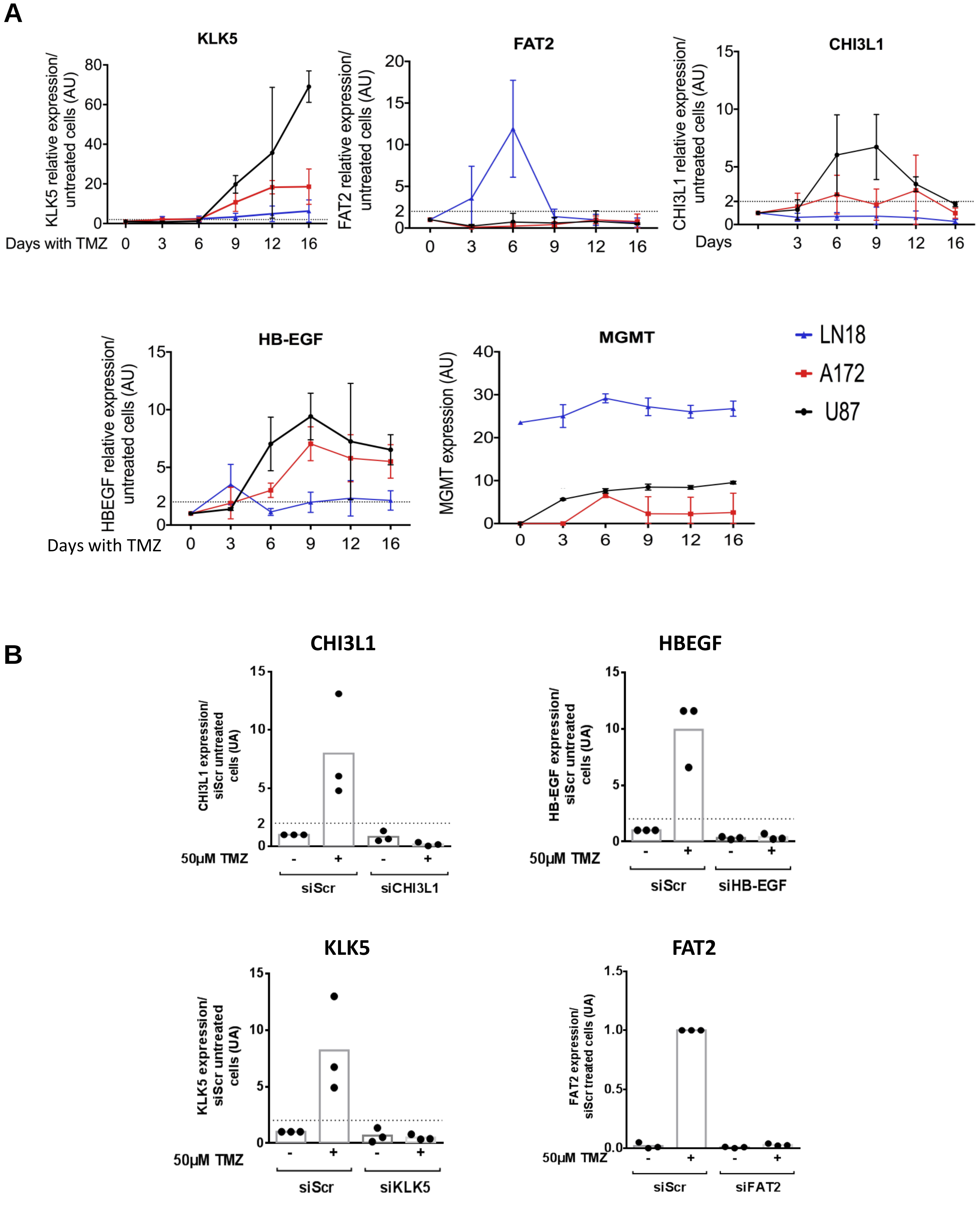

Figure S6 (A) KLK5, CHI3L1, FAT2, HB-EGF and MGMT expression in A172, U87, LN18 cell lines treated respectively by 50, 250 and 350 µM TMZ over 16 days (RT-qPCR).

(B) Expression of CHI3L1, HB-EGF, KLK5, and FAT2 in U251[S] cells knocked down for the respective gene with si RNA (RTqPCR). N=3
